# Effects of a Visual Reaction Training System on Footwork Agility, Reaction Time, and Movement Performance in U12 Badminton Players

**DOI:** 10.3390/s26154660

**Published:** 2026-07-23

**Authors:** Dinh Quang Ngoc, Nguyen Van Phuc, Ting-Hui Chen, Chin-Mou Liu, Lan-Yuen Guo, Yu-Chuan Liu, Kuei-Pin Kuo

**Affiliations:** 1 Institute of Sport Science, Technology and Foreign Affairs, Bac Ninh Sports University, Tu Son 16151, Vietnam; dinhquangngoc.bsu@gmail.com (D.Q.N.); nvphuctdttbn@gmail.com (N.V.P.); 2 Department of Athletic Sports, Chang Jung Christian University, Tainan 711, Taiwan; cph20130120@gmail.com (T.-H.C.); cmliu@mail.cjcu.edu.tw (C.-M.L.); 3 College of Medicine, Kaohsiung Medical University, Kaohsiung 807, Taiwan; yuen@kmu.edu.tw; 4 Office of Physical Education, National Pingtung University of Science and Technology, Pingtung 928, Taiwan

**Keywords:** badminton footwork, agility training, visual reaction training, virtual reality training system (VRTS), youth athletes

## Abstract

**Highlights:**

**What are the main findings?**

- The proposed training system has a significant positive impact on reaction time and movement time in elementary school boys’ badminton.
- The VRTS enables quantitative assessment of agility and coordination by providing objective measurements of movement performance.

**What are the implications of the main findings?**

- The proposed training system is an effective and reliable tool for enhancing the visual reaction and movement skills of elementary school badminton players.
- VRTS intervention in specific footwork training can improve movement speed and reduce reaction time.

**Abstract:**

This study investigated the effectiveness of a Visual Reaction Training System (VRTS) for improving badminton-specific footwork performance in U12 players. Thirty-four participants were randomly assigned to an experimental group or a control group and completed a six-week training intervention. Training effectiveness was evaluated using star-footwork tests under fixed and random conditions, with reaction time and total movement time as outcome measures. Mixed-design ANOVA and ANCOVA were used for data analysis. The results showed that the experimental group achieved significantly greater improvements than the control group in both reaction time and movement performance. A significant group × time interaction was observed for fixed-mode reaction time (F = 12.26, *p* < 0.001, ηp^2^ = 0.28). Significant between-group effects were also found for fixed-mode movement time (F = 204.73, *p* < 0.001, ηp^2^ = 0.87) and random-mode movement time (F = 34.77, *p* < 0.001, ηp^2^ = 0.54). These findings indicate that VRTS-based training effectively enhances agility, visual reaction ability, and footwork execution in young badminton players. The system may serve as a practical tool for both training and performance assessment in youth badminton development.

## 1. Introduction

### 1.1. Research Motivation and Purpose

Badminton is one of the most popular sports in Taiwan and has gained increasing international recognition through the achievements of elite athletes. As participation among youth players continues to grow, the development of effective training and assessment methods has become increasingly important. Agility and rapid visuomotor responses are essential determinants of badminton performance, as players must continuously execute rapid directional changes, accelerations, and perceptual–motor decisions during play. Star-shaped footwork drills are widely used to train these abilities and are considered fundamental for developing movement efficiency in young badminton players [1,2,3,4,5,6]. To enhance training efficiency and standardization, several electronic reaction and agility training systems have been developed. However, most existing systems primarily assess general reaction performance and lack badminton-specific movement patterns. Furthermore, many provide limited integration of training and performance evaluation functions, restricting their ability to monitor sport-specific adaptations. Consequently, there remains a need for a system that combines real-time visual stimuli, badminton-specific footwork training, and quantitative performance assessment.

The Visual Reaction Training System (VRTS) developed by Kuo et al. [7,8] was designed to address these limitations. The system provides LED-based directional cues that simulate movement demands during badminton play while automatically recording reaction time and movement time. This enables objective monitoring of training performance and offers a practical approach for badminton-specific agility training and assessment.

During traditional star drill training, the player stands at the center of the court facing the coach and moves toward one of six directions as indicated by the coach’s gestures. This traditional approach is time-consuming and labor-intensive. Furthermore, this approach does not enable the capturing of data on player reaction time (time taken by the player to move from the center of the court to the designated position). Integrating training equipment with scientific data analysis can make the training process more systematic and efficient [9]. Mancini et al. [10] demonstrated that reliable and precise reaction-time assessment devices enable coaches and educators to accurately monitor athletes’ agility, ensuring that observed performance improvements reflect true development rather than measurement variability. Various badminton-specific training devices have been developed to enhance the efficiency of star drill training, reduce the workload of coaches, and monitor the performance of players during training. For example, Kuo et al. [7] developed a visual reaction movement training system that supports and monitors players engaging in star drill training. Their system improves player agility and enables intelligent monitoring at a low cost. The system features split timing, mode switching for various training types, performance tracking, and feedback provision. These features have been used by researchers to develop a more scientific approach that can improve athletic performance in badminton training [11]. Measurement reliability is a crucial factor in any device or system.

In the proposed training system, six light-emitting diodes (LEDs) are used to signal the target direction to which the player should run [7]. When a specific light is activated, the player runs in the corresponding direction. Because of the system’s sensing mechanism, this training system can measure reaction time, movement time, and total reaction time for a complete run to and from a target point [12,13]. In this paper, reaction time is defined as the interval from the moment an LED is activated until the moment the player leaves the starting point to move toward a target point. The time taken to reach that point is the first movement time, and the time taken to return to the starting point is the second movement time. Adding these times yields the total movement time.

In Taiwan, badminton is a widely practiced sport and has gained increasing attention due to the success of athletes in international competitions. Agility and rapid visuomotor responses are essential for badminton performance, and multidirectional star-footwork drills are commonly used to develop these abilities [14,15]. To facilitate badminton-specific agility training, Kuo et al. [7,8] developed a Visual Reaction Training System (VRTS) that integrates LED-based visual stimuli with star-footwork movement patterns. Previous studies have demonstrated the feasibility and application of the VRTS in badminton training [8]. However, limited evidence is available regarding its effectiveness in improving badminton-specific footwork performance in youth athletes. Furthermore, the relative effects of fixed-pattern and random-pattern training on reaction and movement performance remain unclear. Therefore, the present study aimed to investigate the effects of a six-week VRTS-based star-footwork training program on reaction time, movement time, and total completion time in U12 badminton players. A total of 34 elementary school badminton players were recruited and randomly assigned to either an experimental group or a control group. Following separate six-week training interventions, participants completed identical pre-test and post-test assessments. It was hypothesized that VRTS-based training would significantly improve reaction time, movement time, and overall footwork performance compared with conventional training methods [16].

### 1.2. Definitions

The VRTS was developed by Kuo et al. [7] at National Pingtung University of Science and Technology. The VRTS consists of an industrial-grade programmable logic controller (PLC; Delta Electronics, Taipei, Taiwan) and a human–machine interface (HMI; Delta Electronics, Taipei, Taiwan). Data and HMI information are transmitted via the RS-232 communication protocol, and the internal program is implemented using a Sequential Function Chart (SFC). Signal transmission from infrared sensors and LEDs is achieved through multi-core control cables.

The VRTS also supports wireless communication within an unobstructed range of up to 100 m, operating at a radio frequency band of 2400–2480 MHz. It integrates both training and measurement functions and comprises six LED modules, a programmable controller, and optical sensors.

## 2. Materials and Methods

### 2.1. Experimental Equipment

**Figure 1:**
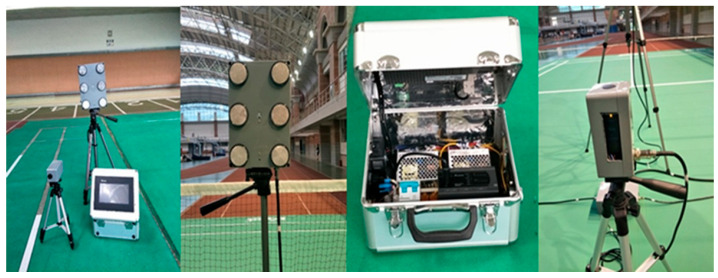
Photograph of the VRTS [8].

The main equipment used in this study was a VRTS (Figure 1), which was designed to administer and monitor badminton-specific footwork agility training through a series of visual stimuli that elicited rapid reactions and agile movement performance. The system utilized LED lights, optical sensing technology, and computer-based programming to establish multiple badminton-specific footwork agility training modules while simultaneously recording various reaction-time-related performance data during each training session. The VRTS integrated LED modules, a programmable logic controller (PLC), and a human–machine interface (HMI). Data transmission was conducted through the RS232 communication protocol, and the internal program architecture was developed using a Sequential Function Chart (SFC). During training, visual guidance was provided through illuminated LED signals positioned at different locations on the court. Participants began from the center point of the court and moved as quickly as possible toward the direction indicated by the visual signal to activate the corresponding optical sensor before returning to the center position to complete the movement cycle. The VRTS simultaneously recorded multiple temporal performance parameters, including reaction time and movement time, during each training session. The performance data collected throughout the training process served as objective feedback for coaches and athletes to evaluate training performance and adjust training focus, methods, and intensity accordingly.

The hardware configuration included infrared sensing modules, LED lights, multi-core control cables, and wireless I/O modules with a maximum transmission range of 100 m. Radio frequency (RF) signal transmission operated within the globally shared 2400–2480 MHz frequency band. The VRTS adopted in this study was adapted from the system developed by Kuo et al. [7]. The reliability and validity of the system were evaluated using the intraclass correlation coefficient (ICC), and the results demonstrated excellent reliability (ICC = 0.95).

**Figure 2:**
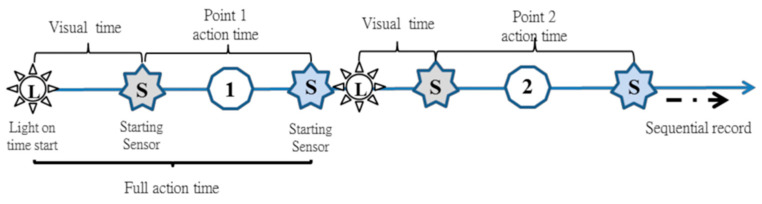
Operation process of the VRTS [8].

The training functions include fixed six-point footwork drills, random six-point footwork drills, and competition simulation training. The VRTS is capable of measuring visual reaction time, directional movement time, total movement time, competition movement time, and T-shaped agility, and provides corresponding outputs in the form of spreadsheets and graphical analyses. Furthermore, the VRTS offers real-time assistance and monitoring during star drill training to enhance players’ agility, thereby meeting the specific demands of badminton training [7]. VRTS was used as the primary training equipment for U12 badminton players in the visual reaction training group (Figure 2). Visual reaction time is defined as the time interval between the onset of a visual stimulus and the initiation of movement, specifically when the participant begins to move away from the starting position (Figure 2). First movement time: First movement time is the time that the participant takes to run from the starting point to a target point (Figure 2). Total movement time: Total movement time is the sum of the visual reaction time and the two movement phases (running toward a target point and returning, Figure 2).

### 2.2. Research Framework

**Figure 3:**
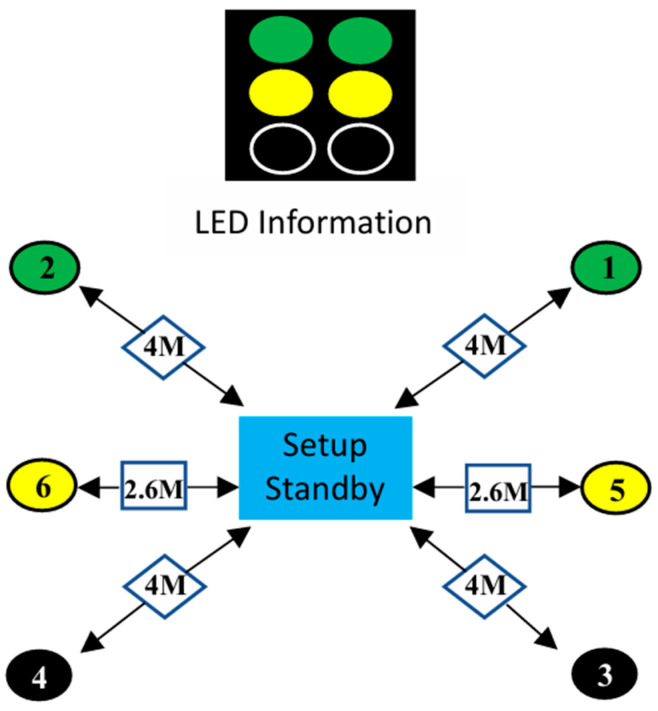
Six-point footwork test layout [8].

In the star footwork test, a visual stimulus panel indicating movement direction was positioned near the center area close to the net at the front of the badminton court. Optical sensors were placed 4 m from the center point at the front-left, front-right, rear-left, and rear-right court positions, while additional sensors were positioned 2.6 m from the center point at the mid-left and mid-right court positions. Participants stood at the center position (30 cm × 30 cm) and were required to move rapidly in the direction indicated by the visual stimulus signals. A schematic illustration of the star footwork test setup is presented in Figure 3. Two different stimulus modes were applied during the test. In the fixed-pattern mode, the LED signals were illuminated sequentially in the order of positions 1, 2, 3, 4, 5, and 6, and participants completed the footwork movements according to the predetermined sequence. In the random-pattern mode, the LED signals were illuminated in a randomized order without a fixed sequence to simulate realistic badminton match situations.

### 2.3. Experiment Date and Locations

Experiments were conducted on 20 July 2020, at three elementary schools in Tainan City, Taiwan (Haidian Elementary School in Annan District, Xinhua Elementary School in Xinhua District, and Yuwen Elementary School in East District).

### 2.4. Participants

This study included 34 members of U12 school boys’ badminton team. All participants had received regular badminton training for more than one year (i.e., more than three sessions per week) and had no history of sports-related injuries within the past year. The mean age of the participants was 10.76 ± 0.98 years, with a mean height of 138.85 ± 6.84 cm and a mean body weight of 31.35 ± 6.45 kg. With parental consent, the participants were randomly assigned to either an experimental group or a control group, with 17 participants in each group.

Sample size estimation. An a priori power analysis was performed using G*Power (Version 3.1.9.7) to determine the minimum required sample size. Assuming a repeated-measures ANOVA (within–between interaction), an alpha level of 0.05, statistical power of 0.80, and a medium effect size (f = 0.25), the required minimum sample size was 34 participants. Therefore, the final sample of 34 participants was considered adequate for the present study.

### 2.5. Experimental Procedures and Description

#### 2.5.1. Research Process

Before the research intervention began, researchers explained the purpose, methods, and procedures of the study to the participants. They then underwent a trial training session with the VRTS (Research-Based Training System) to familiarize themselves with the research environment, equipment, and methods. Researchers carefully checked all instruments to ensure they could record data. Before the formal training intervention, participants practiced the VRTS, and a rest period of at least 48 h was established between the end of the practice and the start of the formal experiment to prevent participant fatigue and potential cumulative effects. Furthermore, the study lasted for 6 weeks, with participants sequentially completing a pre-training test, the training phase (weeks 1–6), and a post-training test. The star footwork ability test was conducted twice before training and twice after training, and the highest individual scores were used as experimental data.

#### 2.5.2. Training Process and Procedures

Both the experimental group (VRTS-guided footwork) and the control group (coach-guided footwork) participated in a six-week badminton footwork training program. During Weeks 1–3, a fixed star footwork pattern (positions 1–6) was employed, whereas during Weeks 4–6, a randomized pattern was implemented. Training sessions were conducted twice per week, with exercises designed to simulate real-match movement demands as closely as possible. Each session lasted approximately 8–12 min and was scheduled outside the participants’ regular badminton training. All sessions were supervised by the same coaching team to ensure consistency in instruction and execution.

Both groups performed star footwork exercises involving six directional movement points commonly used in badminton training. To ensure comparable training load and physical exertion, movement distance, number of directions, total repetitions, and rest intervals were standardized across groups. Specifically, each training session consisted of star footwork drills, with each set involving movement in six directions, as detailed in Table 1.

The primary difference between the two groups was the method of stimulus presentation. The experimental group performed footwork training using the VRTS, which provided either fixed or randomized visual cues depending on the training phase. In contrast, the control group performed the same footwork drills based on traditional verbal or gestural instructions provided by a coach. This control condition was selected because coach-guided instruction is commonly used in footwork training and therefore serves as an appropriate active control for evaluating the effectiveness of VRTS-based training.

Training intensity was not regulated using external load or physiological indicators; instead, it was operationally defined by standardized task structure, movement distance, and number of repetitions, which were consistent across both groups. Participant adherence was monitored throughout the intervention, with attendance rates exceeding 95% in both groups. No injuries or adverse events were reported during the training period.

#### 2.5.3. Testing Protocol

Thirty-four participants completed identical pre-test and post-test assessments before and after the six-week intervention period under standardized testing conditions. All assessments were conducted in the same indoor badminton facility using the same VRTS, equipment, testing procedures, environmental conditions, and evaluators. Before testing, participants completed a standardized warm-up and received identical instructions to ensure consistency.

During each assessment, participants performed both the fixed-pattern and random-pattern badminton star-footwork tasks according to the VRTS protocol. Each test was performed twice, and the shorter completion time was used for statistical analysis. The VRTS automatically recorded visual reaction time, movement time, total reaction time, total movement time, and other agility-related performance variables.

Although the experimental group received VRTS-based agility training and the control group received coach-guided agility training during the six-week intervention period, both groups completed the same standardized VRTS assessments during the pre-test and post-test. Therefore, the only difference between the two groups was the training intervention, whereas all outcome measurements were obtained under identical testing conditions to ensure the comparability and reliability of the recorded results. Although the experimental group received VRTS-based agility training and the control group received coach-guided agility training during the six-week intervention period, both groups completed the same standardized VRTS assessments during the pre-test and post-test. Therefore, the only difference between the two groups was the training intervention, whereas all outcome measurements were obtained under identical testing conditions to ensure the comparability and reliability of the recorded results.

### 2.6. Data Collection and Statistical Analysis

#### 2.6.1. Data Collection

The programmable controller of the training system was used to record the following variables: visual reaction time to LED stimuli, reaction time plus movement time, and total movement time for the star footwork task.

#### 2.6.2. Statistical Analysis

This study adopted a pretest–posttest experimental design to investigate the effects of the training intervention. The independent variable was group assignment (experimental group vs. control group), while the dependent variables consisted of reaction time, movement time, and total time measured under both fixed and randomized testing modes, which served as indicators for evaluating the effectiveness of the training system.

Data were analyzed using SPSS version 24.0 (USA). Descriptive statistics are presented as means and standard deviations. Mixed-design ANOVA was used to examine the effects of group and time on reaction time and movement performance. When significant effects were observed, LSD post hoc comparisons were performed. ANCOVA was subsequently conducted using pretest scores as covariates to compare posttest performance between groups. Statistical significance was set at *p* < 0.05.

## 3. Results

### 3.1. Training Effectiveness: Total Motion Time (Fixed Mode)

**Figure 4:**
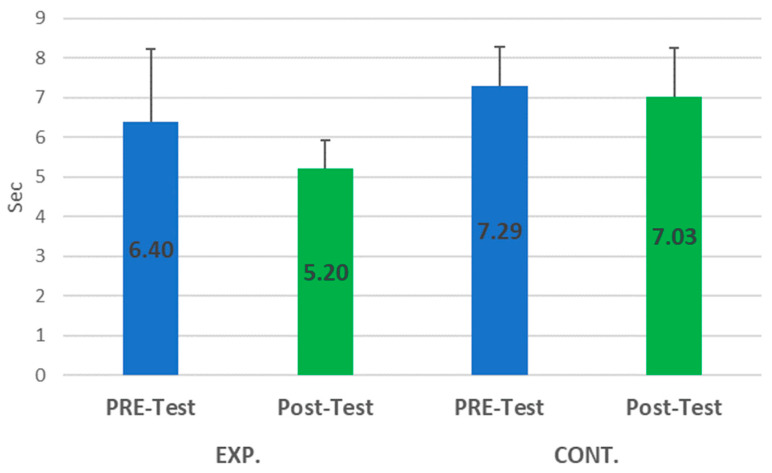
Pre-test and post-test Total Motion Time (fixed mode).

After statistical analysis using a mixed-design approach, Table 2 and Figure 4 indicate that, under fixed-pattern training, no significant interaction effect was observed between group and time. Significant main effects of both group and time were found for total movement time (*p* < 0.05), indicating an overall improvement in total movement time following training. However, differences in total movement time were observed between the two groups.

Therefore, a one-way analysis of covariance (ANCOVA) was conducted, using the pre-test scores as a covariate to examine post-test performance between the two groups. Under the fixed-pattern condition, no significant difference in total movement time was found after controlling for pre-test performance (*p* > 0.05); however, a significant between-group difference was observed in the adjusted model (*p* < 0.05) (Table 3).

### 3.2. Training Effectiveness: Total Motion Time (Random Mode)

**Figure 5:**
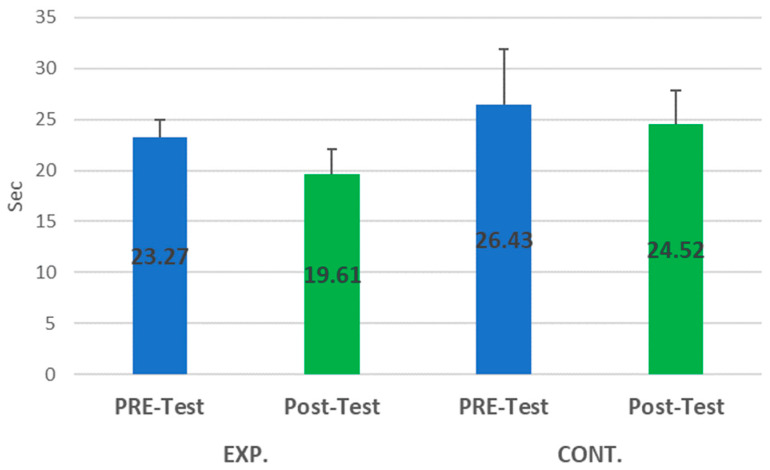
Pre-test and Post-test visual reaction time and movement time (random mode).

After statistical analysis using a two-way mixed-design ANOVA, the results shown in the table indicate that, under randomized training, no significant interaction effect was observed between group and time (Table 4 and Figure 5). The experimental group demonstrated significantly better performance than the control group (*p* < 0.05); however, no significant main effect of time was found (*p* > 0.05). In the subsequent analysis of covariance (ANCOVA), with pre-test scores used as the covariate, significant differences were found in total movement time under the randomized training condition (*p* < 0.05), and a significant between-group effect was also observed (*p* < 0.05) (Table 5).

### 3.3. Training Effectiveness: Reaction Time (Fixed Mode)

**Figure 6:**
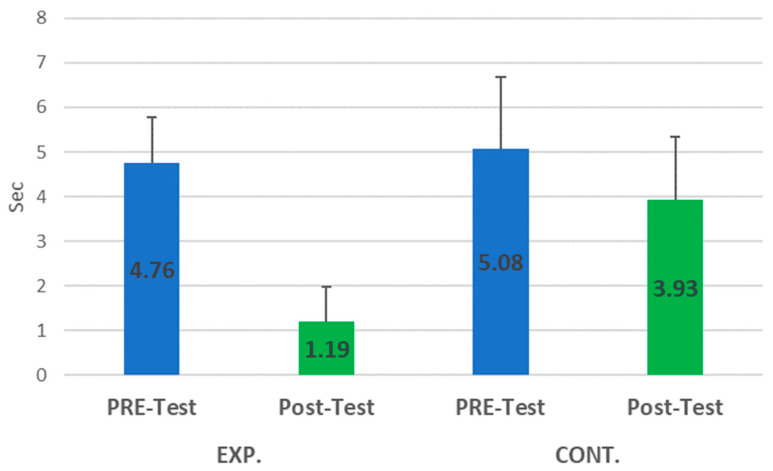
Pre-test and Post-test total reaction time (fixed mode).

Under the fixed-pattern training condition, a two-way mixed-design analysis of variance (ANOVA) on total visual reaction time revealed a significant interaction effect between group and time, as well as significant main effects for both factors (*p* < 0.05; Table 6 and Figure 6). The results further indicated that the experimental group exhibited greater improvement in total reaction time than the control group, with a reduction of 2.02 s.

In addition, analysis of total visual reaction time across six directional points showed significant covariate and between-group effects (*p* < 0.05; Table 7). This finding suggests that, after controlling for pre-test scores, the training intervention led to a significantly greater improvement in total reaction time in the experimental group compared with the control group.

### 3.4. Training Effectiveness: Reaction Time (Random Mode)

**Figure 7:**
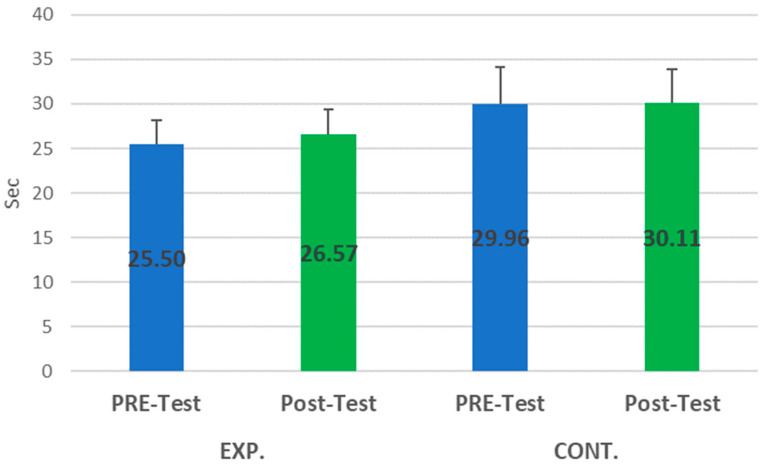
Pre-test and Post-test total reaction time (random mode).

Under the random-pattern training condition, a two-way mixed-design ANOVA on total visual reaction time revealed no significant interaction effect between group and time. However, significant main effects of both group and time were observed (*p* < 0.05; Table 8 and Figure 7). In addition, post-test results indicated that the experimental group exhibited greater improvement in total reaction time than the control group, with a reduction of 1.83 s.

Furthermore, analysis of covariance (ANCOVA) for total visual reaction time across six directional points showed no significant effect of the covariate (*p* > 0.05), whereas a significant between-group difference was observed (*p* < 0.05; Table 9). This finding suggests that, after controlling for pre-test scores, the experimental group demonstrated significantly greater improvement in total reaction time compared with the control group following the random-pattern training intervention.

### 3.5. Results and Discussion

The present study examined the effectiveness of VRTS-based agility training in U12 badminton players. Given that badminton requires rapid offensive and defensive footwork movements, the VRTS was designed to provide sport-specific agility training through system-guided protocols. By integrating visual stimuli with badminton-specific movement patterns, the system served as both a training and assessment tool for monitoring agility performance and training outcomes.

Participants completed a six-week VRTS-based agility training intervention using badminton star-footwork drills as the primary training content. The results demonstrated that, under both fixed-pattern and random-pattern conditions, the experimental group achieved significantly greater improvements in total movement time and total reaction time than the control group, indicating that VRTS-based training can effectively enhance agility-related performance in young badminton players. Although participants were randomly assigned to the experimental and control groups, significant differences were observed in some variables at pre-test. These baseline differences may be attributed to individual variability and the relatively small sample size commonly encountered in youth sport studies. Therefore, the effectiveness of the intervention was interpreted primarily based on pre- to post-test changes and group × time interaction effects rather than baseline comparisons alone. Similar baseline variations have been reported in previous studies involving young athletes and do not necessarily invalidate the interpretation of training-induced adaptations. Although the sample size met the minimum requirement estimated by the a priori power analysis, the relatively small sample may still limit the external validity and generalizability of the findings. Therefore, future studies should recruit larger samples from multiple training centers and different age groups to further validate the effectiveness of the Visual Reaction Training System.

The observed improvements are likely attributable to the visual stimuli incorporated into the VRTS, which simulated shuttlecock trajectories and directional cues encountered during match play. These stimuli required athletes to rapidly identify visual information, make movement decisions, and execute appropriate footwork within a limited time frame, thereby simultaneously challenging perceptual-cognitive and motor processes. Repeated exposure to these sport-specific demands may have enhanced athletes’ ability to process environmental information efficiently and respond more rapidly to changing game situations. These findings are consistent with previous studies demonstrating that visually guided agility training improves reaction time and movement performance in badminton players [17]. Furthermore, perceptual–cognitive abilities such as visual perception, anticipation, and decision-making have been shown to differ according to age and competitive level in adolescent badminton players, emphasizing the importance of developing these abilities during youth training [18]. Recent studies have also shown that agility training integrating visual stimuli with sport-specific movement tasks enhances perceptual-cognitive processing, anticipation, and motor execution in young athletes [19].

The VRTS was developed based on sport science principles and provides objective measurements of movement time, visual reaction time, and motor reaction time. These quantitative data allow coaches and athletes to monitor training progress and evaluate performance changes over time. In addition, the automated design of the system enables standardized training and assessment procedures, thereby reducing potential variations associated with manual observation and recording. Consequently, the system may serve as a practical tool for supporting evidence-based training and performance monitoring in youth badminton programs. The visual guidance component of the VRTS was designed to replicate the perceptual demands of badminton competition. Previous studies have suggested that training environments resembling actual competition conditions facilitate greater transfer of training effects to sport performance [20,21]. The significant improvements observed in reaction time and movement time following the intervention support the effectiveness of sport-specific agility training. Previous research has also indicated that agility training may enhance information-processing efficiency, decision-making ability, and response speed through adaptations of the central nervous system [22]. Therefore, the improvements observed in this study may reflect both perceptual and neuromuscular adaptations induced by VRTS-based training.

The six-point positioning model can be applied to evaluate agility performance across various sports; however, because badminton involves rapid multidirectional movements within a confined court area, this model is particularly suitable for badminton-specific assessment. Moreover, sport science research emphasizes the importance of integrating sport-specific training and testing protocols to improve athletes’ ability to transfer training adaptations to competitive situations [23]. Therefore, the present findings support the use of the star footwork test as an effective method for evaluating training outcomes and agility performance in badminton athletes.

Several limitations should be acknowledged. First, the sample size was relatively small and consisted exclusively of U12 badminton players, which may limit the generalizability of the findings to athletes of different ages and competitive levels. Second, the intervention period was limited to six weeks; therefore, the long-term effects of VRTS-based agility training remain unclear. Future studies should recruit larger samples, include athletes from different age groups and performance levels, and investigate the long-term adaptations associated with VRTS implementation. Additionally, future research may compare VRTS-based training with other agility-training approaches to further determine its relative effectiveness and practical application in badminton training.

## 4. Conclusions and Recommendations

### 4.1. Conclusions

This study investigated the training effects of VRTS-based star footwork training in U12 badminton players. A total of 34 participants were recruited, and a six-week training program was implemented. In terms of training effectiveness, compared with the control group receiving traditional verbal instruction, the experimental group demonstrated significantly greater improvements in total reaction time and movement execution performance under both fixed-pattern and random-pattern intervention conditions.

Fixed-pattern training produced greater improvements during the initial stage of training, which may be attributed to repetitive practice facilitating motor learning and movement automatization. In contrast, random-pattern training enhanced adaptive agility and cognitive–motor integration abilities, with significant between-group differences observed in the analysis of covariance (ANCOVA).

The application of the VRTS for footwork training provides both monitoring and automated training functions. For U12 athletes at the foundational stage of athletic development, the system not only improves reaction speed and familiarity with footwork movements, but also promotes the development of sport-specific agility and visual–motor coordination. These findings are consistent with previous studies examining the application of structured and variable training programs in youth athletes [14,15].

In summary, the VRTS integrates reliable measurement capabilities with effective training mechanisms, demonstrating its dual role as both an assessment and training tool. By providing precise feedback regarding reaction time and movement performance, the system enables coaches and athletes to monitor genuine improvements in motor skill performance rather than superficial changes resulting from measurement error. Therefore, the VRTS may serve as a practical and evidence-based approach for enhancing agility, reaction time, and footwork performance in elementary school badminton players.

### 4.2. Limitations and Recommendations

Several limitations should be acknowledged in this study. First, the participants were limited to male badminton players in Grades 3 to 5 of elementary school. Future studies are encouraged to include participants of different ages, sexes, and athletic backgrounds in order to enhance the generalizability of the findings.

Second, this study examined only a limited number of variables, including total movement time, visual reaction time, movement time, total completion time, and total six-point visual reaction time. Future research may further investigate additional positional points to analyze spatial variability in movement performance.

Third, the intervention program in this study consisted of six weeks of training, conducted twice per week with eight sets per session. Future studies may extend the duration of training and increase both training frequency and training volume to further enhance the effectiveness and adaptive benefits of badminton-specific agility and reaction training.

## Figures and Tables

**Table 1:** Training program and content.

Distribution Item	Week	Week 1–2	Week 3–4	Week 5–6
Exp. group	1	1C × 3n × 2N (r = 7″, R = 90″)	2C × 2n × 2N (r = 8″, R = 120″)	2C × 2n × 2N (r = 10″, R = 120″)
	3	2C × 2n × 1N (r = 7″, R = 120″)	2C × 2n × 2N (r = 10″, R = 120″)	2C × 2n × 3N (r = 10″, R = 120″)
Cont. group	1	2C × 1n × 2N (r = 10″, R = 120″)	1C × 4n × 3N (r = 10″, R = 120″)	3C × 2n × 2N (r = 15″, R = 150″)
	3	1C × 4n × 2N (r = 7″, R = 120″)	3C × 2n × 1N (r = 15″, R = 120″)	2C × 3n × 3N (r = 15″, R = 150″)

C = complete 6 points; n = intragroup count; N = intergroup count; r = intragroup rest; R = intergroup rest, ″= seconds.

**Table 2:** Summary table of total Motion time analysis (fixed mode).

Source	SS	df	MS	F	*p*	${ŋ}_{p}^{2}$
Group	276.66	1	276.66	20.624	0.000 *	0.392
Time	131.394	1	131.394	11.414	0.002 *	0.263
Group × time	12.975	1	12.975	1.127	0.296	0.034
Between						
participants	429.255	32	13.414			
Error	368.371	32	11.512			
Total	1218.655	67	445.955			

* *p* < 0.05.

**Table 3:** Summary table of covariate analysis of total motion time (fixed mode).

Source	SS	df	MS	F	*p*	${ŋ}_{p}^{2}$
Covariate	1.767	1	1.767	0.196	0.661	0.006
Group	204.732	1	204.732	22.661	0.000 *	0.430
Error	271.037	30	9.035			
Total	477.536	32	215.534			

* *p* < 0.05.

**Table 4:** Summary of total movement time (random mode).

Source	SS	df	MS	F	*p*	${ŋ}_{p}^{2}$
Group	272.548	1	272.548	16.507	0.000 *	0.340
Time	6.236	1	6.236	0.863	0.36	0.026
Group × time	3.587	1	3.587	0.496	0.486	0.015
Between						
participants	528.351	32	16.511			
Error	231.165	32	7.224			
Total	1041.887	67	306.106			

* *p* < 0.05.

**Table 5:** Summary table of covariate analysis of total motion time (random mode).

Source	SS	df	MS	F	*p*	${ŋ}_{p}^{2}$
Covariate	11.141	1	11.141	11.481	0.002 *	0.277
Group	34.772	1	34.772	35.834	0.000 *	0.544
Error	29.111	30	0.970			
Total	467.525	32	172.424			

* *p* < 0.05.

**Table 6:** Summary table of total reaction time (fixed mode).

Source	SS	df	MS	F	*p*	${ŋ}_{p}^{2}$
Group	23.4	1	23.400	9.887	0.004 **	0.236
Time	68.04	1	68.04	88.276	0.000 **	0.735
Group × time	12.264	1	12.264	15.992	0.000 **	0.333
Between						
participants	75.738	32	2.367			
Error	24.539	32	0.767			
Total	203.981	67	106.838			

** *p* < 0.01.

**Table 7:** Summary of ANCOVA results for total reaction time in fixed mode.

Source	SS	df	MS	F	*p*	${ŋ}_{p}^{2}$
Covariate	11.141	1	11.141	11.481	0.002 **	0.277
Group	34.772	1	34.772	35.834	0.000 **	0.544
Error	29.111	30	0.970			
Total	75.025	32	46.884			

** *p* < 0.01.

**Table 8:** Summary table of total reaction time (random mode).

Source	SS	df	MS	F	*p*	${ŋ}_{p}^{2}$
Group	31.481	1	31.481	19.013	0.000 **	0.373
Time	9.141	1	9.141	5.941	0.021 *	0.157
Group × time	3.784	1	3.784	2.459	0.127	0.071
Between						
participants	52.985	32	1.656			
Error	49.234	32	1.539			
Total	146.625	67	47.601			

* *p* < 0.05; ** *p* < 0.01.

**Table 9:** Summary of ANCOVA results for total reaction time in random mode.

Source	SS	df	MS	F	*p*	${ŋ}_{p}^{2}$
Covariate	0.051	1	0.051	0.046	0.831	0.002
Group	28.546	1	28.546	26.043	0.000 *	0.465
Error	32.883	30	1.096			
Total	61.480	32	29.693			

* *p* < 0.05.

## Data Availability

The data presented in this study are available on request from the corresponding author. The data are not publicly available to protect the anonymity of the participants.

## References

[1] Lin Y.Z., Tai H.W. (2010). A Biomechanical Analysis of Badminton Footwork for Functional Badminton Shoe Design. J. Phys. Educ. Res..

[2] Cortes N., Quammen D., Lucci S., Greska E., Onate J. (2012). A Functional Agility Short-Term Fatigue Protocol Changes Lower Ex-Tremity Mechanics. J. Sports Sci..

[3] Peng M.L. (2004). Badminton Training Camp.

[4] Baechle T.R., Earle R.W. (2008). National Strength & Conditioning Association. Essentials of Strength Training and Conditioning.

[5] Zemková E. (2016). Differential Contribution of Reaction Time and Movement Velocity to the Agility Performance Reflects Sport-Specific Demands. Hum. Move..

[6] Yildiz S., Ates O., Gelen E., Çırak E., Bakıcı D., Sert V., Kayihan G., Özkan A. (2020). The Relationship between Reaction Time, Agility and Speed Performance in High-Level Soccer Players. Acta Med. Mediterr..

[7] Kuo K.P., Lin H.C., Chen C.H., Tsai H.H., Wu W.T. (2018). Visual Reaction Motion Training System for Badminton.

[8] Kuo K.P., Tsai H.H., Lin C.Y., Wu W.T. (2020). Verification and Evaluation of a Visual Reaction System for Badminton Training. Sensors.

[9] Hsu S.Y. (1997). Biomechanics in Sports.

[10] Mancini N., Moscatelli F., Padova M., Mancini S., Basta A., Guerriero M.A., Pace A., Limone P., Colella D., Grosu V.T. (2024). The Relationship between Reaction Time and Agility Performance in Young Athletes: A Study Using Perception–Action Technological Devices. J. Phys. Educ. Sport.

[11] Chiu H.C. (2010). Quantitative Research and Statistical Analysis: SPSS (PASW) Examples and Explanations.

[12] Lin S.C., Lin Y.F. (2007). Factors Influencing Reaction Time. J. Chin. Phys. Educ..

[13] Chiu M.C. (2013). A Comparison of Reaction Times Among Elementary School Table Tennis Players of Different Skill Levels. Master’s Thesis.

[14] Chou T.S., Lu C.C. (2005). The Application Techniques of Badminton Footwork. J. Coll. Sports.

[15] Chang J.Y., Chang C.H., Chen K.C., Ho K.S., Chen Y.J. (2016). Effects of Six-Week Agility Training on the Footwork and Reaction Ability of Elementary School Badminton Players. Sports Coach. Sci..

[16] Tsai C.L., Huang C.F., Chi S.C. (1997). 3D Biomechanical Analysis of Four Forehand Strokes among Elite Taiwanese Male Badminton Players. J. Chin. Soc. Phys. Educ..

[17] Kuo K.P., Liao C.C., Kao C.C. (2022). Improving Special Ability Performance of Badminton Players through a Visual Reaction Training System. Healthcare.

[18] Wu K.-C., Lee Y.-L., Chen S.-C. (2024). The effects of age and gender and elite levels on perceptual–cognitive skills of adolescent badminton athletes. Front. Psychol..

[19] Hülsdünker T., Riedel D., Käsbauer H., Ruhnow D., Mierau A. (2021). Auditory Information Accelerates the Visuomotor Reaction Speed of Elite Badminton Players in Multisensory Environments. Front. Hum. Neurosci..

[20] Madsen C.M., Karlsen A., Nybo L. (2015). Novel Speed Test for Evaluation of Badminton-Specific Movements. J. Strength Condit. Res..

[21] Chang C.H., Ho K.S., Lin K.C. (2014). Reliability Analysis of Wearable Devices Used in Star Drill Agility Tests. J. Phys. Educ. NPTU.

[22] Schmidt R.A., Lee T.D. (2011). Motor Control and Learning: A Behavioral Emphasis.

[23] Loureiro L.D.F.B., Dias M.O.C., Cremasco F.C., Silva M.G., Freitas P.B. (2017). Assessment of Specificity of the Badcamp Agility Test for Badminton Players. J. Hum. Kinet..

